# Difference in cardiac remodeling between female athletes and pregnant women: a case control study

**DOI:** 10.1186/s12947-022-00280-7

**Published:** 2022-04-13

**Authors:** Loira Toncelli, Lucia Pasquini, Giulia Masini, Melissa Orlandi, Gabriele Paci, Federico Mecacci, Gianni Pedrizzetti, Giorgio Galanti

**Affiliations:** 1 Sport and Exercise Medicine Department of University of Florence, via delle Oblate 4, 50100 Florence, FI Italy; 2 Fetal Medicine Unit, Department for Women and Children Health, Azienda Ospedaliero-Universitaria Careggi, Florence, Italy; 3 Architectural and Engineering Department of University of Trieste, Trieste, Italy

**Keywords:** Athlete’s heart, Pregnancy, Echocardiography, Strain, Preventive cardiology

## Abstract

**Objectives:**

The aim of this study was to detect possible differences in reversible cardiac remodeling occurring in sport training and twin pregnancy. Background: cardiac remodeling occurs in athletes and pregnant women due to training and fetal requirements, respectively. These changes could be apparently similar.

**Methods:**

21 female elite athletes (23.2 ± 5.3 years), 25 women with twin pregnancies (35.4 ± 5.7 years) and 25 healthy competitive female athletes (controls), age-matched with pregnant women (34.9 ± 7.9 years), were enrolled. This latter group was included to minimize the effect of age on cardiac remodeling. All women evaluated through anamnestic collection, physical examination, 12 leads ECG, standard echocardiogram and strain analysis. Sphericity (SI) and apical conicity (ACI) indexes were also calculated.

**Results:**

Pregnant women showed higher LA dimension (*p* < 0.001) compared to both groups of athletes. LV e RV GLS were significantly different in pregnant women compared to female athletes (*p* = 0.02 and 0.03, respectively). RV GLS was also different between pregnant women and controls (*p* = 0.02). Pregnant women showed significantly higher S′ wave compared to female athletes (*p* = 0.02) but not controls. Parameters of diastolic function were significantly higher in athletes (*p* = 0.08 for IVRT and *p* < 0.001 for E/A,). SI was lower in athletes in both diastole (*p* = 0.01) and systole (*p* < 0.001), while ACIs was lower in pregnant women (*p* = 0.04).

**Conclusions:**

Cardiac remodeling of athletes and pregnant women could be similar at first sight but different in LV shape and in GLS, highlighting a profound difference in longitudinal deformation between athletes and pregnant women. This difference seems not to be related with age. These findings suggest that an initial maternal cardiovascular maladaptation could occur in the third trimester of twin pregnancies.

## Background

Athlete’s heart is an adaptive remodeling of cardiac muscle that occurs in response to the increased physiological demands of repetitive overload induced by exercise training [1]. Cardiac modifications in athletes are related to many factors: the level of intensity (low, medium, high) exercise, age, ethnicity, body size, genetic factors and gender [2]. Physical activity has been significantly associated with increasing left ventricular wall thickness and mass index, increasing left and right ventricular end-diastolic volume index, and increasing left and right ventricular indexed stroke volume [1]. Some authors have observed a significant decline in both right and left ventricular ejection fractions at rest in subjects exposed to increasing levels of exercise [3]. This finding might represent the contractile reserve required for maximal stroke volume augmentation during exercise [4].

On the other hand, structural and functional cardiovascular adaptation occurs in pregnancy in response to fetal growth request [5]. These modifications are characterized by an increase of vascular volume, left ventricular mass, cardiac output, heart rate, and a marked decrease of vascular resistance [6–8], which appear in some aspects similar to athletes heart adaptation to exercise training. However, findings on maternal left ventricular systolic and diastolic function are conflicting [9, 10]. Many authors described a significantly enhanced ventricular function in the first two trimesters with a progressive declined thereafter, as well as reduced diastolic reserve and impaired chamber diastolic function at term [11]. Twin pregnancies appear to have a similar remodeling with more pronounced cardiovascular changes than singletons [12, 13]; however, this group of pregnant women has been less investigated.

The aim of this study was to compare two reversible models of cardiac remodeling due to sport training and twin pregnancy.

## Methods

### Subjects

Twenty-one female athletes were selected. They played in the same soccer team (Italian premier league) competing in the topflight of their category for at least 2 years, were trained with the same load 5 times a week, each training lasting ≥2 h, and had a similar lifestyle. The evaluation was performed at the peak of the soccer season, at the Sports and Exercise Medicine Unit of the University of Florence.

Twenty-five women with uncomplicated twin pregnancy referred to the Fetal Medicine Unit of Azienda Ospedaliero-Universitaria Careggi, Florence, were enrolled in the third trimester (32 weeks gestation). Exclusion criteria were multiple es pregnancies. Information regarding maternal demographic and clinical characteristics (age, ethnicity, weight and height, parity, gestational age at the time of examination, maternal obstetric history) were collected.

Twenty-five healthy competitive female athletes, practicing different sports (running, tennis, cycling), were also enrolled at the Sports and Exercise Medicine Unit of the University of Florence. This group was age-matched with pregnant women and, although more heterogeneous than elite athletes’ group, it was introduced in the study in order to reduce the impact of age difference in cardiac remodeling. In fact, these subjects trained about 10.3 ± 5.63 h per week, a training load comparable with the one of elite athletes: this group could then help to possibly differentiate between cardiac remodeling consequent to training and pregnancy.

For all groups, subjects with signs of previous or current cardiovascular disease, positive family history for structural/functional heart disease or sudden cardiac death < 50 years or with poor eco quality on the echocardiogram were not included in the study.

All the participants signed an informed consent form after reading a study-specific information note. Ethical approval was obtained from the local Ethics Commitee.

### Clinical evaluation and anthropometric parameters

All the participants were evaluated through anamnestic collection, physical examination, 12 leads electrocardiogram (ECG) and echocardiogram. Clinical evaluation included measurement of weight, height and blood pressure at rest. Body mass index (BMI) was calculated as “weight (Kg)/ height^2^ (m^2^)” and the body surface area (BSA) was estimated through the DuBois formula [0.007184 x Height (cm) 0.725 x Weight (kg) 0.425] [14].

### Echocardiographic study

Cardiac remodeling was assessed through standard echocardiographic evaluation and speckle tracking in order to calculate global longitudinal strain.

A commercially available Philips iE33 ultrasound instrument medical System (Bothell, WA) equipped with X5–1 transducer was used to perform the echographic examination. A single experienced operator performed all images acquisition. In accordance with the American Society of Echocardiography (ASE) guidelines [15], measurements of the left ventricle (LV) were obtained by M-Mode in parasternal long axis view with the patient at rest in left lateral decubitus. Interventricular septum (IVS) and posterior wall (PW) thicknesses, left atrium (LA) diameter, end-systolic (LVESD) and end-diastolic (LVEDD) ventricular diameters were measured. LV cardiac mass (LVCM) was derived from Devereux formula [16]: *LVCM = 0.80 × 1.04x[(LVEDD + PW + IVS)*^*3*^*- LVEDD*^*3*^*] + 0.6 g*. Wall stress (WStr) was calculated according to La Place formula: *WStr = [SBP x LVEDD/2]/IVS*. Fractional shortening (FS) was calculated using the following equation: LVEDD –LVESD/LVEDD× 100 [17]. Relative wall thickness (RWT) was computed as *2xPW/LVED* [18]. LV end diastolic (LVEDV) and LV end systolic (LVESV) volumes were derived from the apical four and two chamber views using the Biplane Simpson’s method [19–21]; ejection fraction (EF) was calculated as LVEDV –LVESV/LVEDD× 100. Cardiac indices were normalized for BSA. Measurement of mitral and tricuspid annular plane systolic excursion (MAPSE and TAPSE respectively) were performed by anatomical M-mode from the apical four-chamber view and measured in mm between the most basilar position of the annulus in end-diastole and its most apical displacement at end-systole. Diastolic function was evaluated by Doppler analysis in the presence of three stable RR intervals on the ECG and in three different sequential measurements from 4-chamber apical view. Deceleration time (DT), isovolumetric relaxation time (IVRT), early (E) and late diastolic (A) mitral inflow velocities were recorded. Pulsed wave tissue Doppler imaging was performed to obtain the average of the medial and lateral peak systolic velocity (S’) for assessing systolic function, and early diastolic mitral annular velocities (E’) to obtain E/E’ ratio for assessing LV diastolic function. Peak systolic velocity (S) was also computed at the lateral tricuspid annular to evaluate right ventricle (RV) function.

### Speckle tracking echocardiography (STE)

**Fig. 1:**
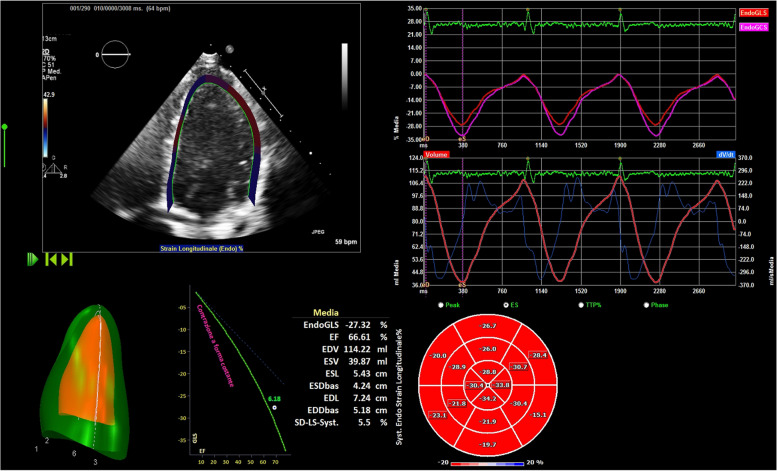
apical four-chamber view for Strain analysis

For STE, images acquisition was performed throughout three consecutive cardiac cycles during breath holds. Standard grey scale images of the RV were obtained from an apical four- chamber view while images of LV were obtained from the apical two-, three- and four- chamber views to derive the global longitudinal strain (GLS) and from the parasternal short axis views, at the basal, mid an apex LV level, to calculate the global circumferential strain (GCS). All the images were transferred to a workstation and analyzed frame by frame using an offline software package (Philips Q-station software - Philips Medical System, Bothell, WA). Endocardial borders were delineated both in the end systolic frame and in the end diastolic frame for each view. The software divided the entire circumference of LV into six equal segments and then tracked the movement of the echocardiographic speckles during myocardial contraction generating myocardial strain curves (Fig. 1).

### Sphericity and apical conicity indexes

**Fig. 2:**
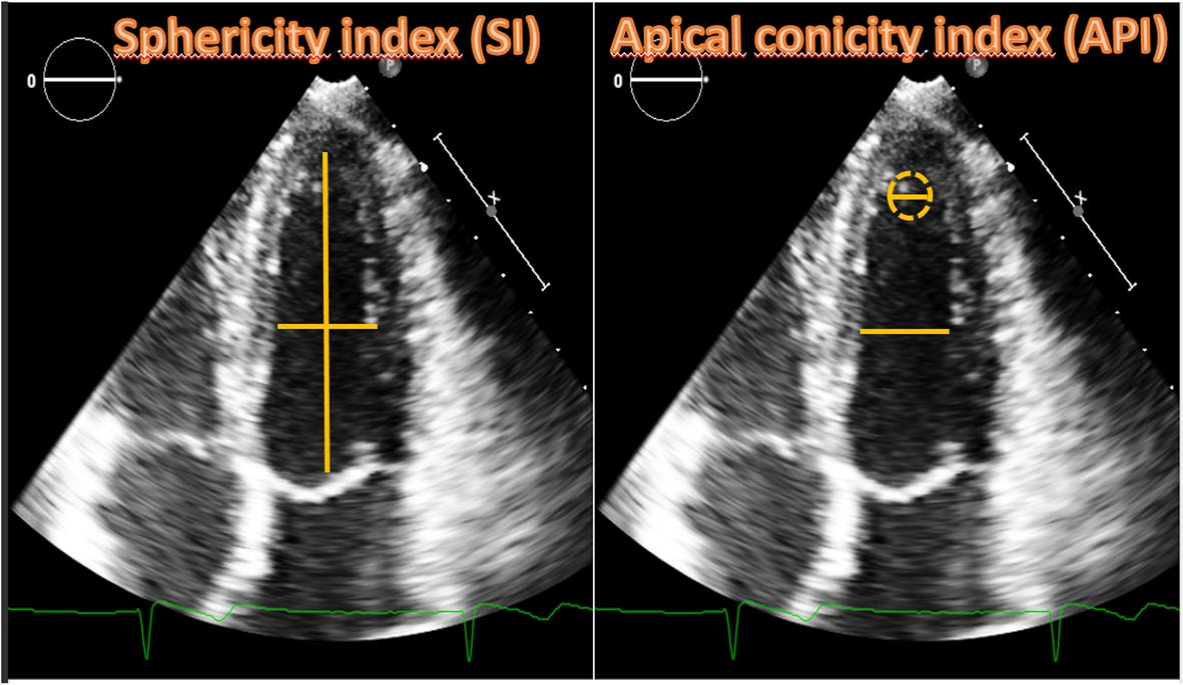
apical four-chamber view for calculation of Sphericity and Apical conicity indexes

Sphericity Index (SI) was calculated as LV basal radial length/longitudinal length, while apical conicity index (ACI) was calculated as apical to short axis ratio [22]: both indexes were measured in end-systole (SIs, ACIs) and end-diastole (SId, ACId) (Fig. 2).

### Statistical analysis

Statistical analysis was performed using the “Statistical Package for Social Sciences” ver. 20 (IBM-SPSS Inc., Chicago, Ill). The variables were reported as mean ± standard deviation. Echocardiographic parameters of the athletes at the peak of the agonistic season were compared with the measurements obtained from pregnant women in the third trimester using Student’s T-test for independent variables. A *p*-value < 0.05 was considered statistically significant.

## Results

Demographic, clinical and echocardiographic parameters of the three groups are shown in Table 1.

**Table 1:** Demographic, clinical and echocardiographic parameters

Parameter	Pregnant women	Athletes	Controls	P (athletes vs pregnant women)	P (pregnant women vs controls)	P (athletes vs controls)
Age (years)	35.4 ± 5.7	23.2 ± 5.3	34.9 ± 7.9	< 0.001	0.64	< 0.001
Height (cm)	164.9 ± 6.5	167 ± 7.1	166.4 ± 5.1	0.195	0.31	0.61
Weight (kg)	67.7 ± 7.85	58.7 ± 5.69	56.2 ± 6.5	0.364	0.06	0.22
BMI (kg/m^2^)	24.94 ± 2.14	20.93 ± 1.04	20.25 ± 1.93	0.004	0.001	0.22
BSA (m^2^)	1.74 ± 0.12	1.66 ± 0.12	1.62 ± 0.11	0.037	0.28	0.28
SBP (mmHg)	115.79 ± 0.39	116.85 ± 0.97	116.4 ± 10.75	0.50	0.08	0.38
DBP (mmHg)	73.61 ± 0.31	72.63 ± 0.80	7.4 ± 7.17	0.29	0.96	0.3
HR (bpm)	70.39 ± 10.35	70.19 ± 14.23	70.7 ± 11.31	0.96	0.26	0.36
IVS (mm)	9.2 ± 0.9	8.8 ± 0.8	8.5 ± 0.7	0.14	0.006	0.97
PW (mm)	8.9 ± 1.3	8.5 ± 0.8	8.3 ± 0.6	0.26	0.06	0.67
LA (mm)	37.4 ± 2.6	32 ± 2.5	31.3 ± 2.2	< 0.001	< 0.001	0.1
LVCM (g)	171.85 ± 29	146,98 ± 20.98	148.42 ± 32.1	0.004	0.01	0.89
iLVCM (g/m^2^)	98.5 ± 14.7	95.5 ± 14.9	91.98 ± 21.37	0.534	0.28	0.74
LVEDD (mm)	48.4 ± 3.1	49.1 ± 9.9	48.3 ± 2.6	0.916	0.26	0.47
LVESD (mm)	30.1 ± 3.3	28.7 ± 4.2	28 ± 3.3	0.263	0.12	0.39
iLVEDD (mm/m^2^)	28.4 ± 2.8	29.8 ± 6.3	28.4 ± 4.6	0.437	0.36	0.52
RWT	0.37 ± 0.06	0.35 ± 0.05	0.35 ± 0.02	0.263	0.71	0.98
LVEDV (mm^3^)	85.5 ± 23	81.6 ± 14.1	77 ± 13.1	0.517	0.16	0.11
LVESV (mm^3^)	35.1 ± 10.7	30.5 ± 5.8	32.1 ± 5.7	0.097	0.36	0.28
Wstr	63.05 ± 10.59	59.36 ± 10.49	60.9 ± 13.8	0.09	0.62	0.35
LVCM/EDV (g/mm^3^)	2.13 ± 0.55	1.80 ± 0.33	1.95 ± 0.43	0.03	0.23	0.24
iLVCM/EDV (g/mm^5^)	1.22 ± 0.32	1.20 ± 0.26	1.21 ± 0.31	0.81	0.94	0.14

Data are expressed as mean ± SD. *BMI* body mass index, *BSA* body surface area, *SBP* systolic blood pressure, *DBP* diastolic blood pressure, *HR* heart rate, *IVS* interventricular septum, *PW* posterior wall, *LA* left atrium, *LVCM* left ventricular cardiac mass (i: indexed); *LVEDD* LV end-diastolic diameter (i: indexed), *LVESD* end-systolic diameter, *RWT* relative wall thickness, *LVEDV* left ventricular end-diastolic volume, *LVESV* left ventricular end systolic volume, *Wstr* parietal systolic stress

A significant difference in mean age (*p* < 0.001) and LA dimension (*p* < 0.01) was found between pregnant women and both athletes. LVEDV, LVESV, iLVCM and wall thickness were similar in the three groups. A significant difference between pregnant women, and both athletes and control groups was identified for LVCM (*p* = 0.004 and *p* = 0.01, respectively), which disappeared when normalized for BSA. All the groups showed RWT below the value of 0.42, and Wstr value was higher in pregnant women without reaching statistical significance.

Table 2 shows LV and RV systolic and LV diastolic function. LV systolic function parameters were slightly different in pregnant women compared to the other groups, however, only GLS was significantly different when compared to athletes (*p* = 0.02). Parameters of diastolic function were significantly higher in athletes and control group compared to pregnant women (*p* = 0.008 and *p* = 0.01 for IVRT in pregnant women vs athletes and controls, respectively; *p* < 0.001 between athletes and pregnant women for E/A).

**Table 2:** Systolic and diastolic parameters

Parameter	Pregnancy	Athletes	Controls	p (athletes vs pregnant women)	p (pregnant womne vs controls)	p (athletes vs controls)
EF (%)	58.9 ± 4.2	62.1 ± 6.3	63.5 ± 3.4	0.08	0.06	0.36
FS (%)	37.7 ± 5.1	41 ± 3.7	39.6 ± 4.7	0.57	0.65	0.51
MAPSE (mm)	18.5 ± 1.8	19.2 ± 1.7	19.8 ± 2.7	0.26	0.06	0.44
E/A	1.28 ± 0.29	2.2 ± 0.7	1.7 ± 0.4	< 0.001	0.05	0.32
IVRT (s)	67 ± 9.9	75.6 ± 9.2	74.6 ± 8.3	0.008	0.01	0.48
DT (m/s)	169 ± 24	182 ± 27.9	181.4 ± 25.4	0.13	0.12	0.35
E/E’	6.1 ± 1.6	6 ± 0.7	5.76 ± 0.82	0.76	0.18	0.47
S (LV) (m/s)	10.2 ± 1.7	10.2 ± 1.1	10.8 ± 1.6	0.98	0.14	0.18
TAPSE (mm)	23.7 ± 4.2	23.9 ± 3	23.5 ± 2.7	0.86	0.74	0.19
S (RV) (m/s)	14.2 ± 2.6	12.5 ± 1.5	13.1 ± 1.6	0.02	0.09	0.81
**LV** GLS (%)	−23.7 ± 2.9	−26.5 ± 4.1	− 26.5 ± 2.8	0.02	0.05	0.68
**LV** GCS (%)	−28.6 ± 4.3	− 30.7 ± 4.9	−27.4 ± 3.9	0.16	0.42	0.06
RV GLS (%)	−29.8 ± 6.3	−27.4 ± 3.9	−26.7 ± 5.8	0.03	0.02	0.54

Data are expressed as mean ± SD. *LV* left ventricle, *RV* right ventricle, *EF* ejection fraction, *FS* fractional shortening, *MAPSE* mitral annular plane systolic excursion, *E/A* early (E)/late diastolic (A) mitral inflow velocities, *IVRT* isovolumetric relaxation time, *DT* deceleration time, *E/E*’ ratio between E and early diastolic mitral annular velocities (E’) assessed with TDI, *S* average of the medial and lateral peak systolic velocity assessed with TDI, *TAPSE* tricuspid annular plane systolic excursion, *GLS* global longitudinal strain, *GCS* Global circumferential strain

With regard to RV systolic function, pregnant women showed significantly higher S’ wave compared to female athletes (*p* = 0.02) but not controls, and no significant difference in TAPSE; RV global longitudinal strain was significantly lower in pregnant women compared to both athletes and control group (*p* = 0.03 and 0 = 0.02, respectively).

**Fig. 3:**
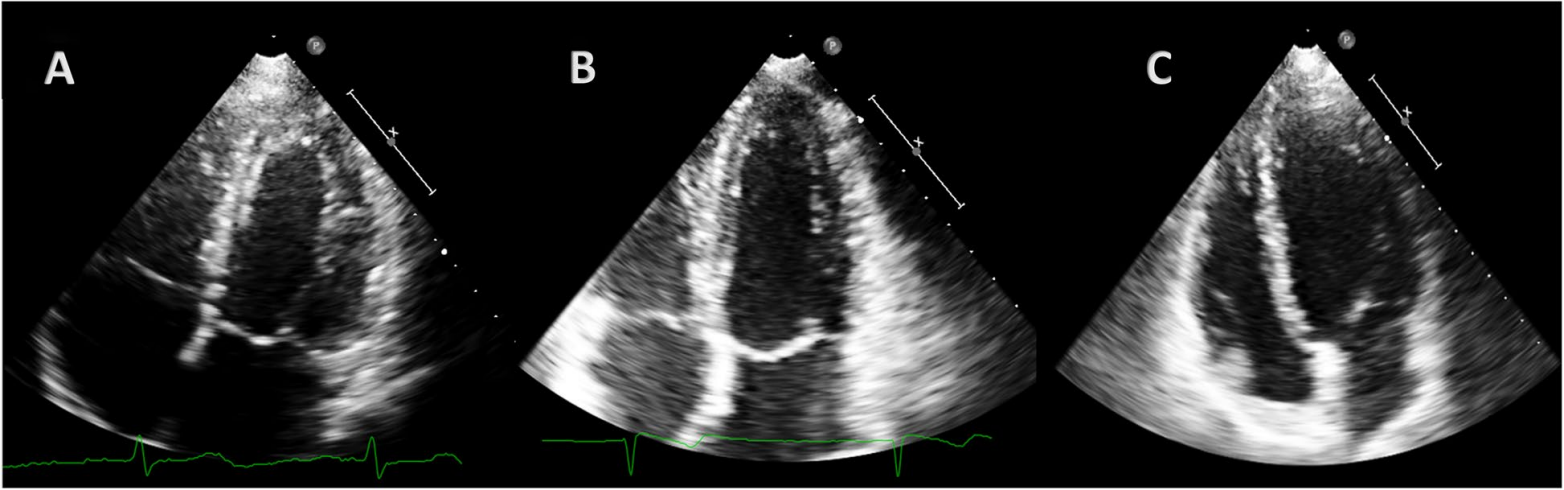
**A** apical four-chamber view of LV in a pregnant woman. **B** apical four-chamber view of LV in an elite athlete. **C** apical four-chamber view of LV in a woman included in the control group

Table 3 shows left ventricular sphericity and conicity indexes. Both athletes and controls showed lower values of SI compared to pregnant women, both in diastole (SId, *p* = 0.01 for both groups) and systole (SIs, *p* < 0.001 and *p* = 0.005 for athletes and control groups, respectively), while ACI was lower in pregnant women (ACIs, *p* = 0.04 and *p* = 0.05 for athletes and control groups, respectively). Figure 3 shows the difference in left ventricular shape in the three groups in an apical four-chamber view.

**Table 3:** Sphericity and conicity indexes

Parameter	Pregnant women	Athletes	Controls	p (athletes vs pregnant women)	p (pregnant women vs controls)	p (athletes vs controls)
LDd	78.25 ± 7.94	76.05 ± 7.34	77.90 ± 6.59	0.08	0.1	0.56
LDs	67.16 ± 9.98	64.87 ± 7.67	65.87 ± 8.63	0.02	0.04	0.42
RDd	46.01 ± 6.09	36.90 ± 5.45	37.45 ± 3.69	< 0.001	< 0.001	0.41
RDs	37.35 ± 3.8	29.40 ± 5.29	31.26 ± 4.32	0.01	0.03	0.36
SId	0.56 ± 0.07	0.49 ± 0.07	0.49 ± 0.63	0.01	0.01	0.9
SIs	0.54 ± 0.05	0.48 ± 0.06	0.49 ± 0.17	< 0.001	0.005	0.74
ApDd	19.63 ± 5.72	15.89 ± 3.09	16.96 ± 7.32	0.21	0.32	0.65
ApDs	13.98 ± 4.12	12.21 ± 2.27	12.91 ± 2.64	0.72	0.84	0.79
ACId	0.38 ± 0.08	0.41 ± 0.09	0.40 ± 0.31	0.23	0.43	0.42
ACIs	0.33 ± 0.07	0.38 ± 0.11	0.37 ± 0.28	0.04	0.05	0.47

Data are expressed as mean ± SD. *LDd* longitudinal diastolic diameter, *LDs* longitudinal systolic diameter (ricontrollare abbreviazione), *RDd* radial diastolic diameter, *RDs* radial systolic diameter (ricontrollare abbreviazione), *Sid* diastolic sphericity index, *Sis* systolic sphericity index, *ApDd* apical diastolic diameter, *ApDS* apical systolic diameter, *ACId* diastolic apical conicity index, *ACIs* systolic apical conicity index

## Discussion

Cardiovascular system undergoes important morphological and functional adaptations that lead to an apparent similar heart remodeling both in athletes and in pregnancy. The aim of the present study was to compare these two types of remodeling considered both reversible and physiological. Cardiovascular assessment of the subjects included in the study was performed when the stress load for the cardiovascular system was at its maximum, i.e. at the peak of competitive season for female athletes [23] and in the third trimester for pregnant women [8].

Similar cardiovascular adaptation was observed between athletes and control groups, although mean age between the two groups was different. On the other hand, both groups showed differences in cardiac morphology, function and strain analysis when compared to pregnant women. Since the control group was age-matched with twin pregnancies, it is possibile that pregnancy itself could lead to a different cardiac remodeling compared to training. Of note, the difference between mean age of athletes and pregnant women was approximately 12 years, which is considered a short amount of time, not able to determine a significant impact on cardiac remodeling [24, 25].

### Morphology

The main differences identified between both groups of athletes and pregnant women were in left atrial dimension and left ventricular cardiac mass. Elite athletes often show a moderate dilatation of the left atrium [26, 27], which is confirmed by our findings. Our data showed that this morphologic modification is particularly increased in pregnant women: this is probably related to the progressive rise in preload due to plasma expansion during pregnancy [28] and not a sole effect of a higher mean age [24]; in fact, women in the control group showed atrial dimension similar to athletes, despite mean age in this group was comparable to pregnant women.

Pregnant women also showed a higher LVCM [7, 11]. Some authors reported that cardiac mass increased by 35% over the course of pregnancy compared with 25% reported in elite athletes on intensive training [23]. The reasons for this difference could be the persistent increase in volume overload in pregnancy compared to the intermittent though repetitive overload in exercise training.

### Functional parameters

RWT was below 0.42 in all groups, defining an eccentric LV remodeling [29]. Wstr was within the normal range, suggesting an adequate myocardial adaptive response to the hemodynamic changes [7, 30, 31] although values ​​tended to be higher in pregnancies.

Regarding diastolic function, our data are in accordance with literature. Our athletes had a trans-mitral flow velocity, expressed by the E/A ratio, > 2: athletes’ diastolic function is normal or even super-normal at rest due to the lower contribution of LA active contraction and possibly also to the lower velocity of the untwisting of the left ventricle (as showed by the increased IVRT) [32]. Pregnancy instead is characterized by hemodynamic changes that have contrasting influence on LV diastolic function [13, 33]. The reduction in systemic vascular resistance is expected to facilitate LV emptying and may lower LV filling pressures; on the other hand, the increase in circulating blood volume and HR would increase trans-mitral flow velocities. The effect of these two alterations on LV diastolic function, LA and LV filling pressures may be variable. Our study showed a markedly lower E/A ratio (*p* > 0.001) and a shorter IVRT in pregnant women (*p* = 0.008) compared to the athletes. These findings suggest a drop in diastolic function due to reduced ventricular compliance, a hypothesis already formulated by other authors in studies both on single [34] and twin [35] pregnancies.

### Strain analysis

Speckle tracking has a greater role in evaluating left contractile function compared to “classical” EF estimation [36, 37] because it is less influenced by the preload, as it happens in pregnancy [11]. Strain speckle tracking echocardiography, in fact, can detect subtle changes in cardiac deformation [11] and is a fine-tune, highly reproducible, operator-friendly method for quantification of left ventricular function [37]. We observed that GLS was significantly lower in the third trimester of pregnancy compared to athletes and control groups, although the systolic function analyzed through classical load-dependent parameters (EF, S) did not differ within groups. Many studies agree in not considering the decrease in longitudinal deformation as indicator of an adverse effect of pregnancy on global left cardiac performance [38], however, studies have mainly concentrated on singleton pregnancies. Nevertheless, we hypothesize that this finding could be suggestive for an initial deterioration in myocardial function in the third trimester of twin pregnancies, in which the haemodynamic changes are more marked than single ones. To our knowledge, this difference could not be consequent of the age difference between groups, as 12 years are not sufficient to significantly worsen strain values [25]. In fact the control group showed similar results compared to elite athletes.

Longitudinal strain by speckle-traking echocardiography was initially designed to evaluate left ventricular function. More recently, it has been introduced to assess RV performance, although this evaluation remains technically challenging due to RV unique structure and physiology. Limited data have been published regarding RV longitudinal function in pregnancy, while athletes’ RV strain has been more investigated. Some studies report that intense endurance exercise was associated with a mildly reduced RV global and regional longitudinal strain. This finding could be considered a “physiological” consequence of intensive exercise conditioning and could be included in the context of cardiovascular changes of the so-called “athlete’s hearth” [39, 40]. In contrast, other studies, which concentrated on post-exercise RV function, reported a significant decrement in RV compared to LV function, showing that RV is more intensively fatigued after endurance exercise due to a greater wall stress and work [41]. Furthermore, Lewicka-Potocka et al. 2020, reported that strenuous exercise declines not only RV systolic but also diastolic function [42], In our study, strain values of RV were significantly lower in the athletes compared to pregnant women, despite being within the normal range in all groups. This result could reflect an impairment in the athletes’ RV function in accordance with the studies reported above.

### LV geometry

An important parameter to take into account when applying a speckle-tracking technique to athletes’ heart is LV geometry: indeed, changes in mechanical loading of the LV are often accompanied by a change in LV shape. Regional longitudinal wall curvature and thickness are important determinants for the amplitude and shape of the transmural distribution in passive end-diastolic fiber stress and strain [43]. A 3D geometric analysis in the assessment of the athletes’ hearth highlighted that LV long axis length and apical curvatures are increased in marathoners’ hearts, with normal sphericity indexes [44].

Healthy pregnancy is universally accepted as a state of physiological adaptation to a protracted volume overload state with preserved intrinsic myocardial contractility/relaxation. In the first half of pregnancy, preload is higher because of increased venous return, whereas afterload decreases due to reduction of peripheral vascular resistances, leading to a change of LV geometric shape. At first, myocardial contractility increases improving myocardial deformation indices which reach peak values in the second trimester [44, 45], while, at term, cardiac hypertrophy is not completely balanced by a further increase in LV size and cardiac afterload rises; a more spherical shape of the left chamber and a reduction of GLS are observed. GLS in fact is primarily determined by the vertically arranged subendocardial fibers; therefore, deformation indices are more susceptible to alterations in load conditions [44, 46].

One of the major factors defining a physiologic response to chronic hemodynamic stress is the adherence to an elliptical LV shape, as opposed to an increase in LV sphericity, and the maintenance of a normal LV mass/volume ratio. Deviations from a normal match of geometry and muscle mass can cause increase in wall stress and myocardial oxygen demand, resulting in decreased LV function. In our study a more spherical and less conical shape of LV was observed in pregnant women compared to female athletes, resulting disadvantageous for ventricular function; these data were also confirmed by decreased GLS values.

### Strenghts of the study

The inclusion of a control group, age-matched with pregnant women, allowed to identify modifications due to pregnancy and differentiate them from age-related cardiac alterations.

### Limitations of the study

We aknowledge that the sample size of our study is small, particularly for pregnant women; however, the high risk of pregnancy complications in twin pregnancies, such as preeclampsia, preterm delivery, fetal growth restriction, gestational diabetes or fetal death, gives reason for the difficulty in obtaining a more numerous study group.

## Conclusions

The data provided are of value in differentiating heart load-related remodeling. Starting from the concept that both pregnancy and training lead to a physiological cardiac adaptation, our findings suggest that an initial maternal cardiovascular worsening of LV performance could occur in the third trimester, and this seems to be a peculiar characteristic of twin pregnancies. Younger female athletes, undergoing a similar load increase on cardiovascular system, and age-matched female competitive athletes, in fact, show a remodeling, without maladaptive characteristics. Our study could be an early finding to further investigation in future.

## Data Availability

The datasets generated and/or analyzed during the current study are not publicly available due to the fact that they contain information that could compromise the privacy of research participants; data are available from the corresponding author on reasonable request. The data that support the findings of this study are available on request from the corresponding author GG. The data are not publicly available due.

## Abbreviations

ECG: Electrocardiogram
BMI: Body mass index
BSA: Body surface area
SBP: Systolic blood pressure
DBP: Diastolic blood pressure
HR: Heart rate
IVS: Interventricular septum
PW: Posterior wall
LA: Left atrium
LVCM: Left ventricular cardiac mass (i: indexed)
LVEDD: LV end-diastolic diameter
LVESD: End-systolic diameter
RWT: Relative wall thickness
LVEDV: End-diastolic volume
LDESV: End systolic volume
Wstr: Parietal systolic stress
LV: Left ventricle
RV: Right ventricle
EF: Ejection fraction
FS: Fractional shortening
MAPSE: Mitral annular plane systolic excursion
IVRT: Isovolumetric relaxation time
DT: Deceleration time
TAPSE: Tricuspid annular plane systolic excursion
GLS: Global longitudinal strain
GCS: Global circumferential strain
LDd: Longitudinal diastolic diameter
LDs: Longitudinal systolic diameter
RDs: Radial diastolic diameter
RDs: Radial systolic diameter
ApDd: Apical diastolic diameter
ApD S: Apical systolic diameter
Sid: Diastolic sphericity index
Sis: Systolic sphericity index
ACId: Diastolic apical conicity index
ACIs: Systolic apical conicity index
